# Propofol-dexmedetomidine versus propofol-ketamine for endoscopic retrograde cholangiopancreatography sedation

**DOI:** 10.6026/973206300220665

**Published:** 2026-02-28

**Authors:** Nancy Ekka, Aditya Singh Chauhan, Brajesh Kumar, Sourabh Shrivastava

**Affiliations:** 1 Department of Anaesthesiology, Gajra Raja Medical College, Gwalior, Madhya Pradesh, India; 2 Consultant Anaesthesiologist, Bansal Hospital, Sagar, Madhya Pradesh, India

**Keywords:** Endoscopic retrograde cholangiopancreatography (ERCP), procedural sedation, propofol, dexmedetomidine, ketamine, hemodynamic stability, recovery time, psychomimetic effects

## Abstract

Deep sedation during elective endoscopic retrograde cholangiopancreatography (ERCP) ensures immobility, success and stability in prone positions, yet propofol monotherapy risks hypotension, bradycardia and respiratory depression. Therefore, it is of interest to compare propofol-dexmedetomidine (PD) versus propofol-ketamine (PK) in 120 ASA I-III adults undergoing ERCP. PD provided superior hemodynamic stability (MAP fluctuation 7.9±3.1% vs. 13.8±4.7%, p=0.001), reduced propofol needs (178±46 mg vs. 241±59 mg, p=0.001) and faster recovery (11.8±3.4 vs. 17.2±5.1 min, p=0.001). Endoscopists rated PD sedation quality higher, with fewer interruptions and better patient tolerance during the procedure. PD advances ERCP sedation by offering safer, more efficient alternatives to common regimens, minimizing complications while optimizing outcomes in high-risk prone procedures.

## Background:

ERCP is a complicated endoscopic operation, which needs deep sedation or general anesthesia to attain patient immobility, reflex suppression and endure long-term prone positioning of the patient [1]. The process has the intrinsic risks of hypoxia, aspiration and hemodynamic instability caused by peritoneal stimulation, biliary colic and the necessity of the use of repeated contrast injections [2]. The use of propofol has been the sentinel of the global ERCP sedation due to the rapid onset, short period context-sensitive half-time and superior recovery data [3]. Its application as a single agent however is linked to dose-related respiratory depression, hypotension and no analgesia and frequently requires high bolus dose to overcome intense stimulation during sphincterotomy or stone removal [4]. To eliminate the need of these constraints, different adjuvants have been used together with propofol. Dexmedetomidine is a very selective 2- adrenergic agonist, which achieves cooperative sedation, analgesia and sympatholysis without any major respiratory depression [5, 6]. It has reduced propofol requirements and still allowed spontaneous ventilation, which has been shown in upper gastrointestinal endoscopy and colonoscopy [7, 8]. By the mechanism of NMDA receptors antagonism, ketamine offers a deep level of analgesia along with the finalization of airway reflexes and breathing motion, which is opposed to the hypotension caused by propofol by stimulating the sympathetic system [9]. Propofol combined with ketamine (ketofol) has become popular in emergency department procedural sedation and paediatric anesthesia [10, 11]. Nonetheless, ketamine is linked with psychomimetic effect, increased oral secretions and delayed recovery, which were not desirable in adults undergoing ERCP [12]. Despite the individual study of both combinations as an alternative to propofol alone or midazolam alone, direct head-to-head comparison propofol-dexmedetomidine versus propofol-ketamine in the ERCP per se is limited. The small sample size, non-blindedness, or the use of fixed-ratio mixtures of these trials are limitations [13, 14]. Therefore, it is of interest to find out which sedation combination-Propofol-Dexmedetomidine (PD) or Propofol-Ketamine (PK) offers better hemodynamic stability and endoscopist rated quality of sedation.

## Materials and Methods:

This is a prospective randomized, double-blind, parallel-group, controlled trial that was carried out in March 2022 to February 2023. The sample size was based on initial data with a mean difference in MAP fluctuation of 5% (SD 7, 0.05 = 90%), a sample size of 56 patients per selected group was needed. A group size of seventy patients was used to accommodate the possibility of drop outs, but only 60 patients each group was eventually randomized following exclusion. Included were adult patients aged 18 70 years, ASA physical status I -III, BMI 18 35 kg/m^2^ and elective ERCP. The exclusion criteria included emergency presentation, known allergy to the study medication, severe bradycardia (HR of less than 50 bpm), second or third degree heart block, severe valvular heart disease, decompensated heart failure, pregnancy, psychiatric disorders, chronic opioid or benzodiazepines, predicted difficult airway (Mallampati class IV with limited neck movement). The randomization was done in blocks of ten and was done by an independent statistician using the computer generated random timing. The concealment of allocation was provided through sequentially numbered, opaque, sealed envelopes. A dedicated pharmacist who would not be involved in patient care made study drugs in the same 50-mL syringes and this ensured patient, endoscopist and anaesthetists administering sedation, as well as recovery room nurses, were blinded. All the measures were done in the prone position where the standard monitoring was done (ECG, NIBP every 3 min, SpO_2_, EtCO_2_ over nasal prong adapted to prone position and Ramsay Sedation Score/MOAA/S). The supplemental oxygen was provided at 4 L/min using modified face mask.

## Group PD (Propofol-Dexmedetomidine):

Dexmedetomidine loading dose of 0.6 µg/kg/10 min and then infused at 0.2-0.7 µg/kg/h with control of Ramsay Sedation Score of 4-5.

## Group PK (Propofol-Ketamine):

Ketamine 0.4 mg/kg intravenous bolus during 2 minutes and saline infusion during the same time at the same rate in order to preserve blinding. Propofol in both groups was used with a starting dose of 0.5-1 mg/kg bolus and constant 10-25mg increments after every 1-2 minutes to ensure that Modified Observer Assessment of Alertness/Sedation (MOAA/S) score remained at 1-2. Boluses and infusions were terminated during the procedure (last contrast injection or stent insertion). Primary outcomes were Percentage change in heart rate and mean arterial pressure at baseline (calculated as maximum deviation /baseline x 100).

[1] Rated indigenous sedation quality by endoscopist (4-point scale: excellent, good, fair, poor)

[2] Post-procedure full recovery time (MOAA/S = 5 and can give date of birth)

Secondary outcomes consisted of total dose of propofol, time of procedure, incidence of respiratory depression (SpO_2_ <90 percent more than 10 s or during apnea), hypotension (MAP less than 60 mmHg), bradycardia (HR less than 50 bpm), excessive salivation, psychomimetic effects (hallucinations, agitation, nightmares during recovery) and patient satisfaction (0 to 10 numeric rating scale at 2 hours post-procedure). The statistical analysis was conducted with the SPSS version 27.0. Shapiro-Wilk test was used to test normality. Continuous variables are presented in the form of mean +SD or median (IQR); categoric variables are presented in the form of numbers and percentages. Such tests as independent t-test or Mann Whitney U test and chi-square/Fisher exact test were selected as suitable. Intragroup hemodynamic changes were analyzed using repeated-measures ANOVA and p < 0.05 was taken as the significant value.

## Results:

A total of 120 patients completed the study with no dropouts or protocol violations. Baseline characteristics including age, gender, BMI, ASA status, indication for ERCP and procedure duration were similar between groups (Table 1). The PD group demonstrated significantly better hemodynamic stability and lower propofol requirements (Table 2). Adverse events and patient satisfaction are shown in (Table 3).

## Discussion:

This is a randomized blind trial that has revealed evident propofol-dexmedetomidine combination superiority over propofol-ketamine as a sedative during ERCP. The most vivid observation made was the significantly better hemodynamic stability in the PD group with about 43% and 39% reduction in mean arterial pressure and heart rate variability respectively. This indicates the sympatholytic effect of dexmedetomidine and its capability to inhibit sympathetic reactions to peritoneal manipulation and scope changes [15]. Conversely, the sympathomimetic action of ketamine, despite being applicable to counteract the propofol-induced hypotension in certain studies, caused more significant hemodynamic variabilities in this group. Decxmedetomidine acting as a strong sedative with opioid- sparing actions through alpha2 receptors in locus coeruleus, the decrease by 26 percent in the total consumption of propofol is not surprising [6]. Reduced dosage of propofol directly correlated with the reduction in respiratory depression episodes, which is of paramount safety importance in prone-positioned patients without certain airway protection. The PD group had almost 5 minutes faster recovery which can probably be attributed to the fact that ketamine was not subjected to the protracted redistribution period and psychomimetic onset. The fact that psychomimetic effects were seen almost in one-fifth of patients administered with low doses of ketamine, but not in the PD group (which had no hallucinations, no agitation and no unpleasant dreams), can be viewed as the key clinical advantage [12, 16]. Endoscopists gave higher ratings of the quality of sedation with dexmedetomidine by far, which may be because of easier operating conditions, as the patient is not moving around and better able to endure extended periods in the prone position. The PD group has higher scores in patient and endoscopist satisfaction, which adds to the clinical significance of these objective changes. Bradycardia necessitating atropine was found to be higher with dexmedetomidine, although that difference was not significant and there was not even a patient with severe bradycardia or asystole. This safety is acceptable profile with respect to bigger trials with the same loading and maintenance doses [17]. Limitations are single-center type, ASA IV patients are excluded and the duration of procedures is relatively short (mean less than 45 minutes), which cannot represent complex therapeutic ERCPs and longer than 90 minutes. The cost-effectiveness of the intervention, long-term cognitive outcomes and performance in high-risk groups should be assessed in future research.

## Conclusion:

The propofol-dexmedetomidine combination provides superior hemodynamic stability, requires significantly lower propofol doses and ensures a shorter, smoother recovery compared to propofol-ketamine during elective ERCP. It also improves sedation quality while minimizing respiratory complications and completely eliminating psychomimetic effects. These advantages, coupled with higher patient and endoscopist satisfaction, establish propofol-dexmedetomidine as the preferred sedation regimen for adult ERCP procedures.

## Figures and Tables

**Table 1:** Baseline demographic and procedural characteristics

**Parameter**	**PD Group (n=60)**	**PK Group (n=60)**	**p-value**
Age (years)	52.4±13.8	54.1±12.6	0.492
Gender (Male/Female)	34/26	31/29	0.713
BMI (kg/m^2^)	26.8±4.3	27.3±4.1	0.526
ASA status (I/II/III)	18/32/10	16/30/14	0.614
Indication (stone/strict/malignancy/other)	38/12/6/4	36/14/5/5	0.892
Procedure duration (min)	42.6±14.3	44.1±15.2	0.584

**Table 2:** Primary outcomes: hemodynamics, sedation quality and recovery

**Parameter**	**PD Group (n=60)**	**PK Group (n=60)**	**p-value**
Heart rate fluctuation (%)	11.8±4.2	19.4±6.1	<0.001
MAP fluctuation (%)	7.9±3.1	13.8±4.7	<0.001
Total propofol dose (mg)	178±46	241±59	<0.001
Endoscopist sedation quality (excellent)	54 (90.0%)	48 (80.0%)	0.032
Recovery time to MOAA/S = 5 (min)	11.8±3.4	17.2±5.1	<0.001

**Table 3:** Secondary outcomes and adverse events

**Parameter**	**PD Group (n=60)**	**PK Group (n=60)**	**p-value**
Respiratory depression (SpO_2_ <90% >10 s)	4 (6.7%)	13 (21.7%)	0.019
Airway intervention required	2 (3.3%)	8 (13.3%)	0.048
Hypotension requiring vasopressor	3 (5.0%)	11 (18.3%)	0.024
Bradycardia requiring atropine	5 (8.3%)	1 (1.7%)	0.207
Excessive salivation requiring suction	2 (3.3%)	10 (16.7%)	0.014
Psychomimetic effects (any)	0 (0%)	11 (18.3%)	0.002
Patient satisfaction score (0-10)	8.9±1.0	7.7±1.4	<0.001
Endoscopist satisfaction score (0-10)	9.3±0.8	8.6±1.2	<0.001
